# Accelerated and enhanced osteointegration of MAO-treated implants: histological and histomorphometric evaluation in a rabbit model

**DOI:** 10.1038/s41368-018-0008-z

**Published:** 2018-03-22

**Authors:** Xin Li, Haiyang Xu, Baodong Zhao, Shuai Jiang

**Affiliations:** 1 Department of Oral Implantology, the Affiliated Hospital of Qingdao University, Qingdao, China; 2 School of Stomatology, Qingdao University, Qingdao, China; 3 Department of Oral Implantology of Zibo Central Hospital, Zibo, China

## Abstract

Microarc oxidation (MAO) has become a promising technique for the surface modification of implants. Therefore, the aims of this study were to further quantitatively and qualitatively evaluate the osteointegration abilities of MAO-treated and smooth surface (SF) implants in vivo and to investigate the areas in which the superiority of MAO-treated implants are displayed. In a rabbit model, a comprehensive histomorphological, osteogenic, mineralizational, and integrative assessment was performed using light microscopy, fluorescence microscopy, confocal laser scanning microscopy, and radiographic analyses. Compared with the SF groups, the MAO-treated groups exhibited more active contact osteogenesis, as well as distant osteogenesis, under fluorescence examination, the mineral apposition rate was found to be greater for all of the MAO-treated implants, and the osteointegration index (OI) value was greater in the MAO-treated groups at different times. In conclusion, the calcium-rich amorphous layer created by MAO provided a better environment for osteointegration, with more active contact osteogenesis, a more rapid mineral apposition rate and greater OI values.

## Implants: A better basis for building bone

A pretreatment procedure that creates a porous, mineral-coated surface on dental implants leads to superior integration into bone. Micro-arc oxidation (MAO) can be used to apply thick coatings enriched in phosphorus and calcium onto titanium implants. Researchers led by Baodong Zhao of The Affiliated Hospital of Qingdao University in China have demonstrated that this complex three-dimensional surface offers a supportive environment for bone growth. Zhao and colleagues examined the integration of MAO-treated titanium implants into the femurs of live rabbits relative to implants with smooth surfaces. After 12 weeks, MAO promoted more active bone formation and mineral deposition, and the researchers observed that bone cells readily penetrated the porous surface of these implants to enable extensive skeletal integration. These results suggest that this procedure might meaningfully improve the outcome of dental procedures in the clinic.

## Introduction

Titanium (Ti) implants have been extensively applied clinically as excellent biomaterials for dental and orthopaedic areas^1^ due to their desirable characteristics, including good mechanical properties, corrosion resistance, and biocompatibility.^2^ Despite the exceptional biocompatibility of Ti and the high success rate in practical applications,^3^ poor osteointegration persists in many cases.^4^ Improving the osteointegration of Ti implants remains a key topic in implantology.

Implant surface properties, such as chemistry, surface, topography, wettability, and charges, have been shown to play important roles in implant osteointegration.^5^ It thus appears that surface properties, such as surface chemistry and topography, influence biological reactions, including protein adsorption, cell–surface interaction, and cell–tissue organization at the interface between the bone and implant, leading to improved osteointegration with the host bone.^6^ Surface treatment techniques for Ti implants have been widely reported in the literature to boost osteointegration during the early healing phase.^7^ Several surface modification methods have been developed over many years, including sandblasting,^8,9^ plasma spaying,^10^ the sol-gel method,^11^ electrophoretic deposition^12^ and microarc oxidation (MAO).^13–15^

Research on the biological response of bone tissues to implants has demonstrated that the MAO process is one of the techniques that improves the osteointegration of Ti implants.^16,17^ MAO is an electrochemical surface modification technique using high voltages (several hundred volts) to fabricate porous and thick oxide coatings on metals^18^ and to incorporate phosphorus (P) and calcium (Ca) ions into the surface layer.^19^ One attractive property of MAO is its ability to create a macro-porous and firmly adherent TiO_2_ film on Ti surfaces and consequently to modify the surface chemistry and topography.^20^ Morphology observations have revealed that implants treated with MAO could form a porous multipore topography because of the discharges occurring under high potentials and adequate hydroxyl groups, resulting in a hydrophilic surface.^21^ The porous multipore topography would provide a better material environment for cell bonding, survival, and differentiation and could have a large effect on the rate of osteointegration.^22^ Moreover, the porous and rough surface fabricated by MAO could provide a larger contact area at the sample-medium interface when soaking in culture medium, which can facilitate the adsorption of proteins and be favourable for promoting cell responses.^23^ The porous structure is considered to be beneficial for osteointegration because it allows bone in-growth and subsequently results in mechanical interlocking between the implant and bone.^24^ In situ-grown MAO coatings with nanostructured bioactive oxide layers exhibit admirable interfacial adhesion, provide superior corrosion resistance performance^25,26^ and possess high porosity levels and porous microstructures that are beneficial for tissue growth. Moreover, an MAO-treated layer deposited onto a sandblasted surface showed a favourable combination of roughness and residual stresses, as well as better bioactivity.^20^ Teng et al.^27^ have demonstrated that MAO coatings treated at higher voltages (180 and 200 V) exhibited effects on early osteoblast mineralization. Felgueiras et al.^28^ recently showed that the anodic surfaces created by MAO processes manifested increasing osteoblast attachment and differentiation (ALP production and mineralization), as well as osteointegration.

In addition, MAO treatment can promote an increase in surface bioactivity through the incorporation of ionic species, such as of Ca, P and magnesium (Mg), which are elements natively present in the bone, into the newly formed TiO_2_ layer.^28–30^ Osteoconductive Ca phosphate coatings stimulate bone healing, leading to rapid bonding of implants.^31^ Ca and P incorporation has been demonstrated to improve the interaction between anodized Ti implants and the surrounding bone and to have a direct effect on cellular responses, such as osteoblast proliferation and differentiation, gene expression and the overall osteointegration process.^28,29,31,32^ Ca-incorporated implants exhibited stronger removal torque values and intensively mineralized osteoids on the surfaces of implants^33^ and acceleration of Ca phosphate formation. Implants containing P can improve initial attachment, spreading, and differentiation of osteoblast; Ca ions can be adsorbed onto the surface.^34^ Ribeiro et al.^35^ utilized MAO as a tool to develop multifunctional Ca-rich surfaces, and they found that the amorphous layer, which was rich in Ca, improved fibroblast viability and metabolic activity, as well as osteoblast adhesion (preferentially adhering to the Ca-rich amorphous oxide layer, rather than to crystalline-rich regions).

Polarized MAO-treated Ti is known to be osteopromotive and cytocompatible in vitro.^36^ Most of the studies, however, have focused on the molecular mechanisms underlying the osteointegration of MAO-modified implants in vitro, and appropriate in vivo models investigating the osteointegrative ability associated with bone formation and resorption around MAO-treated Ti implants have been less often demonstrated. Further detailed studies including in vivo experiments are needed to elucidate the effects of MAO coating on bone responses histologically and histomorphometrically. In this study, to obtain insights into the osteointegrative ability of implants, Ti implants were modified by MAO, which is favourable for apatite deposition and osteoblast behaviour,^37^ and their surface characteristics were evaluated. We inserted MAO-treated implants into rabbit femurs, fluorescent staining was applied to trace cellular activity, and the proliferation and calcification of bone cells were labelled under fluorescence microscopy, while confocal laser scanning microscopy (CLSM) was employed for accuracy measurements of osteogenesis. The main aim of this paper was to investigate the in vivo cellular and tissue responses, such as differentiation, proliferation, integration, and mineralization, quantitatively and qualitatively.

## Results

### Surface Analysis

**Figure 1:**
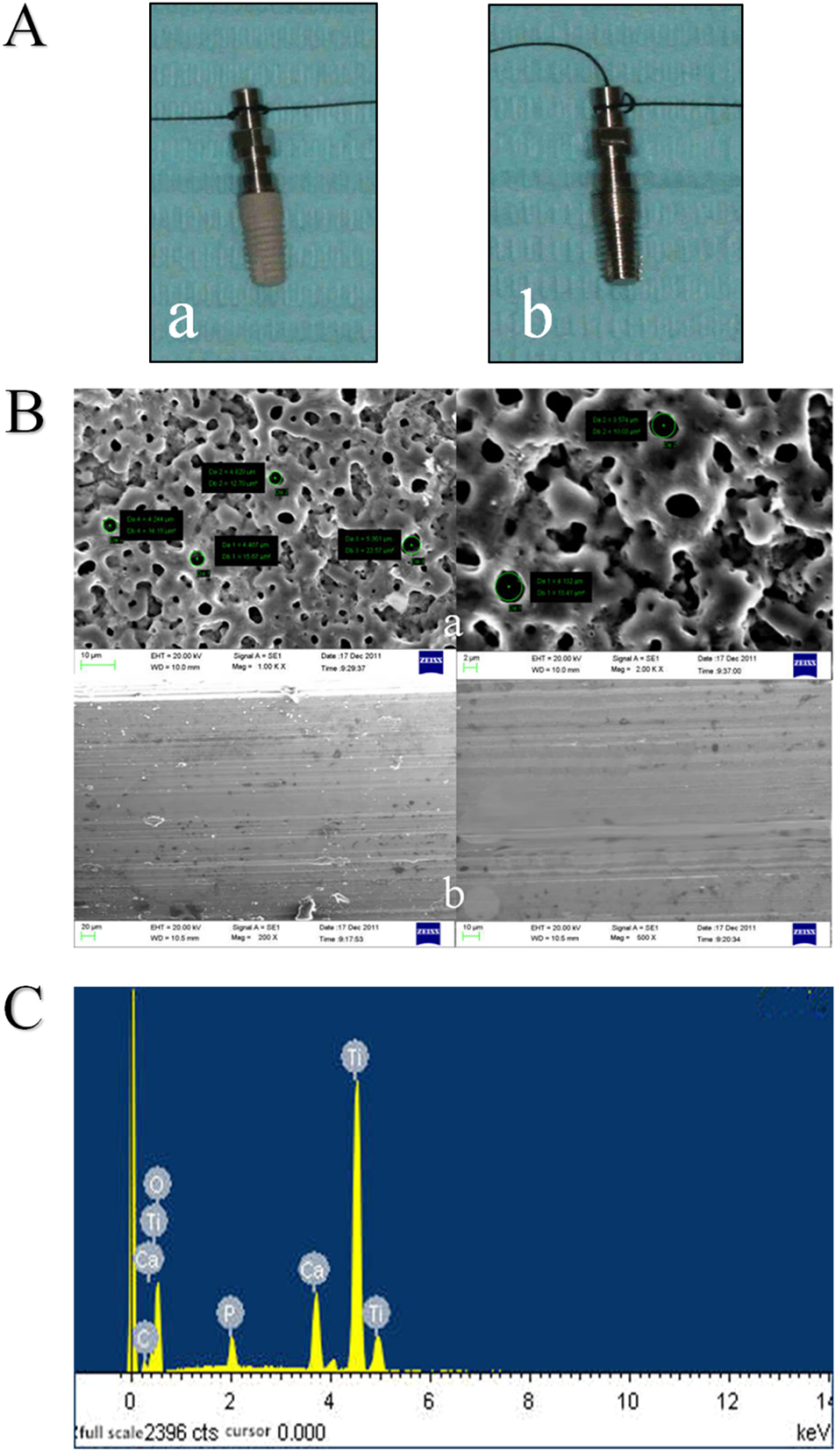
Surface analysis of MAO-treated implant and SF implant. A: **a** MAO-treated implant, **b** SF implant. B: SEM images of the surface topography. **a** MAO-treated implant, **b** SF implant. C: EDS spectra from the surface of the MAO-treated implant. EDS energy dispersive X-ray analysis, MAO microarc oxidation, SF smooth surface

MAO-treated and SF implants are shown in Fig. 1a. Implants treated with MAO exhibited a rough, flat and light greyish surface, while a smooth and polished surface could be observed on implants without MAO treatment.

Regarding the surface roughness analysis, implants treated with MAO formed a porous crateriform topography, which resulted in increased surface roughness (Table 1).

**Table 1:** Ra of MAO-treated and SF implants

Group	MAO-treated	Smooth surface
Ra/μm	1.28±0.04	0.44±0.01
*P*-value	*P*<0.05	

*MAO* microarc oxidation, *Ra* surface roughness, *SF* smooth surface

Regarding SEM morphology, SEM images of the MAO and SF groups are shown in Fig. 1b. In the MAO-treated group, porous topography with the pore diameter ranging from a submicron scale to ~7 µm on the surface of the implants was detected under SEM. The surface morphology seemed like a circular crater with upheaved edges and holes connected with each other, and tiny cracks existed on the film. SF implants exhibited relatively smoother surfaces with scratches in the same direction.

Energy dispersive X-ray analysis (EDS) results revealed that P and Ca were incorporated into the surface layer after modification with MAO technique. The elements on the film consisted of Ti, O, Ca, P, etc (Table 2). The atomic concentration of Ca was 4.55% ± 0.16%, while that of P was 2.16% ± 0.16%, and the Ca/P ratio was 2.12 ± 0.16. The EDS spectrum is shown in Fig. 1c.

**Table 2:** Percentages of elements on the surface film of MAO-treated implants

Elements	Atomic percentage	Weight percentage
C K	4.36	2.16
O K	66.93	44.13
P K	2.14	2.75
Ca K	4.59	7.60
Ti K	21.98	43.41
Amount	100	100

*MAO* microarc oxidation

### Fluorescence examination

#### Week 4

**Figure 2:**
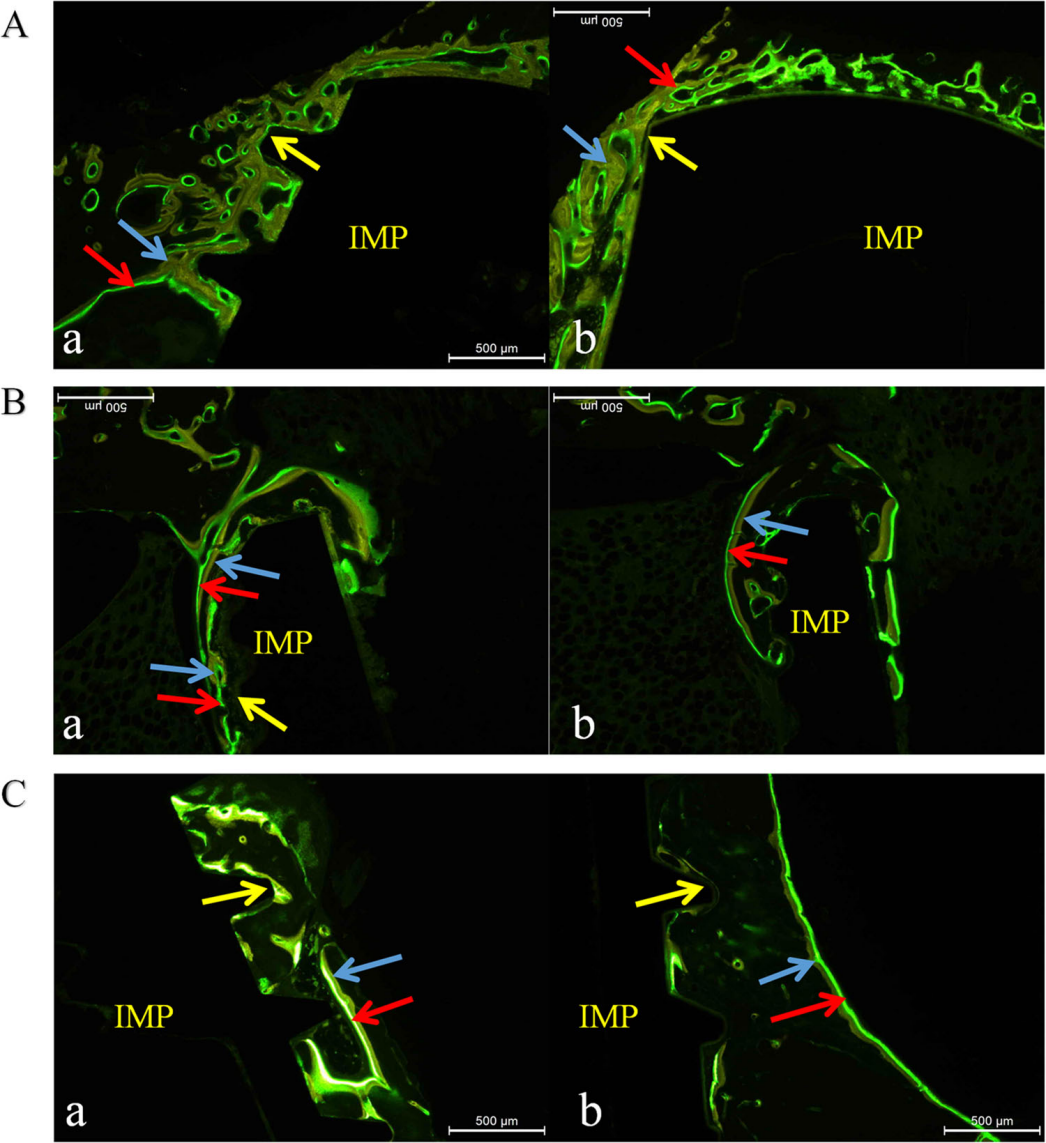
Fluorescent observation at different times. The blue arrows in the figures represent fluorescent yellow labelled by tetracycline, the red arrow represents fluorescent green treated with calcein, and the yellow arrow indicates bone-to-implant interface; IMP means implant. A: Week 4. **a** SF group, **b** MAO-treated group; B: Week 8. **a** SF group, **b** MAO-treated group; C: Week 12. **a** SF group, **b** MAO-treated group. MAO microarc oxidation, SF smooth surface

The SF group (Fig. 2A (a)) showed a relatively narrow bi-colour fluorescence band compared with the MAO-treated group; the yellow band, which was relatively wider, contacted closely with the implant, and the ratio of yellow to green was greater than 1. Osteogenesis presented multicentrically without sheet distributions. Most green bands labelled by calcein were at a distance towards the implant, exhibiting distant osteogenesis.

The MAO group (Fig. 2A (b)) manifested a wide bi-colour fluorescence band, compared with the SF group. The proportion of green bands was greater than in the SF group, in which multiple yellow and green bands appeared. Green bands labelled by calcein appeared both on the surface and towards the implant, exhibiting distant osteogenesis, as well as contact osteogenesis.

#### Week 8

The SF group (Fig. 2B (a)) showed a relatively narrow and irregular fluorescent band, compared with MAO-treated group at the same time. A number of multicentrically fluorescent areas transformed into a continuous bi-colour fluorescent band, indicating that multiple independent bone growth units began to form a whole. The most obvious phenomenon was that a portion of the fluorescent areas began to move away from implant, indicating that osteogenesis was basically completed in these areas. Green bands developed in two directions: towards and away from the implant.

The MAO-treated group (Fig. 2B (b)) manifested a faster osteogenesis rate than in the SF group with a wide and regular fluorescent band. Osteogenesis began to slow as indicated in the smaller proportion of green bands. Almost all of the green bands were located outside the yellow bands, revealing a gradual lateral progression of osteogenesis centred on the implant.

#### Week 12

In the SF group (Fig. 2C (a)), bi-colour areas appeared, and the proportion of green bands decreased significantly, suggesting fairly slow activity of bone growth. Fluorescent bands moved farther away from the bone–implant interface. There was still a large number of bi-colour bands contacting with the implant with green bands inside, and it was more obvious than in the MAO-treated group at the same time, indicating that osteointegration continued.

In the MAO-treated group (Fig. 2C (b)), that the fluorescent band at the contact area had almost disappeared indicated that osteointegration had nearly completed; the fluorescent band was farther from the bone–implant interface than in the SF group, and the proportion of green bands decreased significantly. The most important difference was that almost all of the green bands were located outside of the yellow bands, revealing that osteogenesis developed outwards from the implant.

### Confocal laser examination and the mineral apposition rate (MAR) assessments

**Figure 3:**
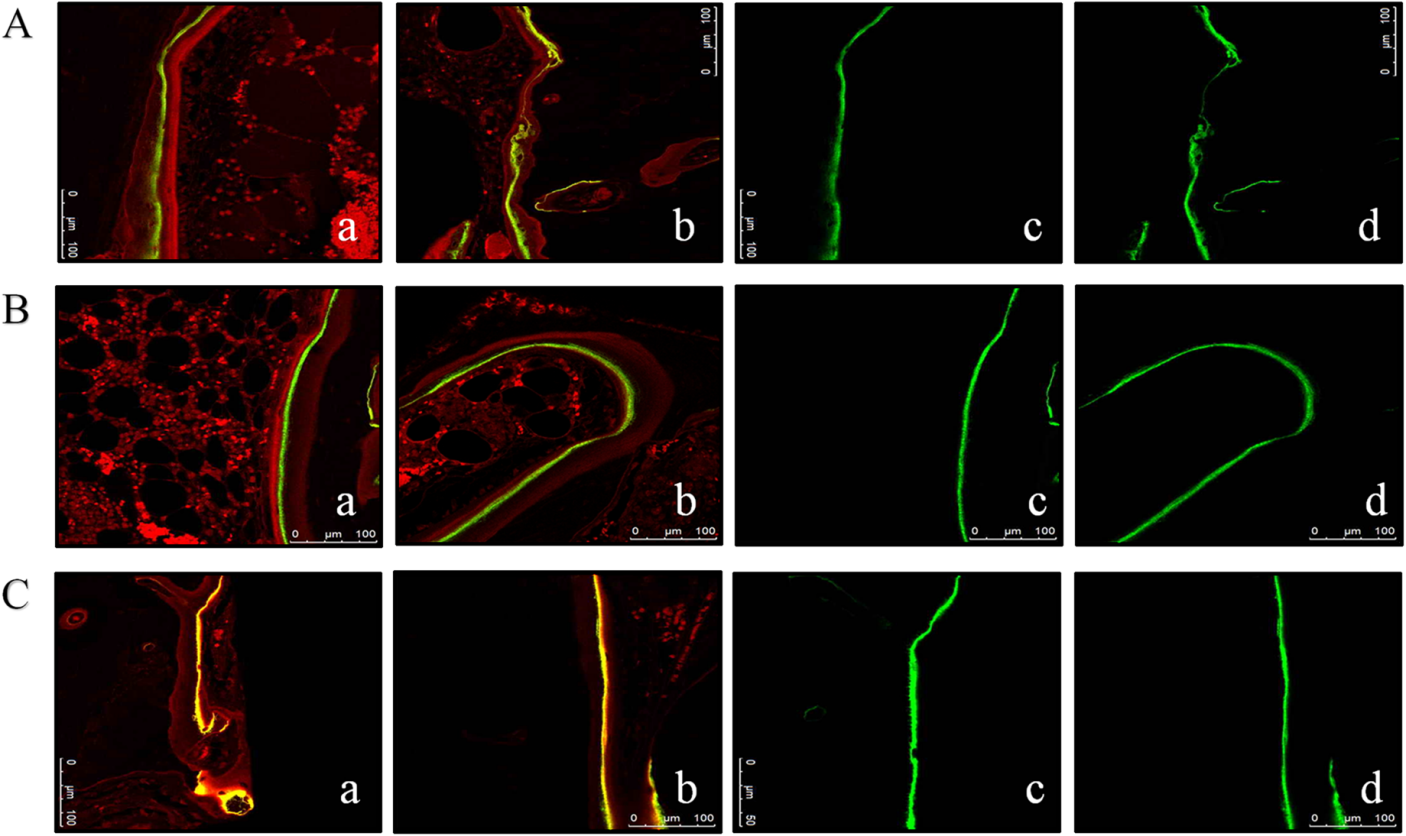
Confocal laser scanning microscopy (CLSM) images at at different times. A: Week 4; B: Week 8; C: Week 12. Activated by green laser: **a** SF group, **b** MAO-treated group. Fluorescent green strip selected by Image-pro plus software, version 6.0: **c** SF group, **d** MAO-treated group. MAO microarc oxidation, SF smooth surface

**Figure 4:**
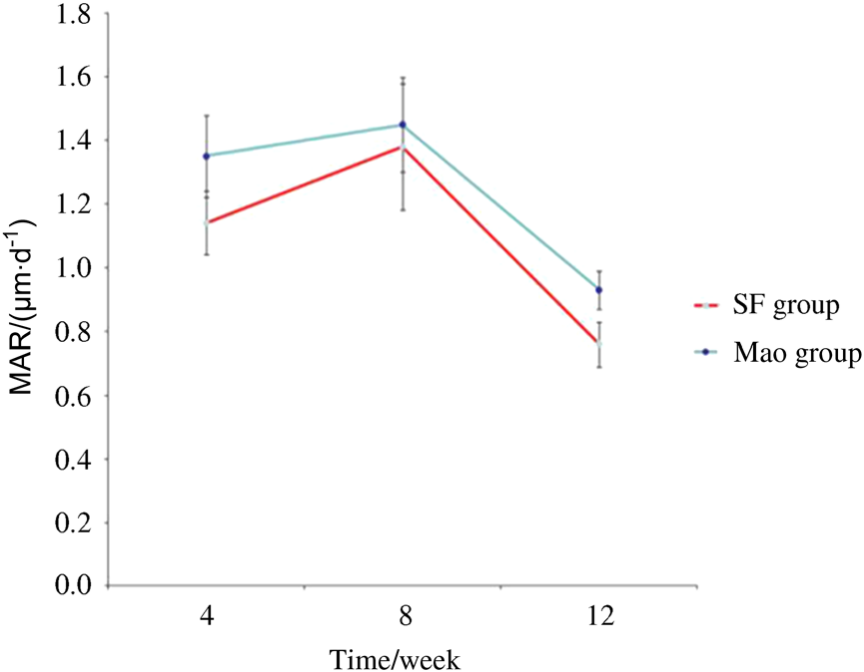
Line chart presenting MAR in the SF and MAO-treated groups at each observation period (μm·d^−1^). MAO microarc oxidation, SF smooth surface

After excitation by green laser at wavelengths of 538–544 nm, areas labelled by calcein could be clearly distinguished from areas marked by tetracycline; fluorescent green strips, which represented mineral apposition zones, were selected for width measurement. CLSM images at weeks 4, 8, and 12 are shown in Fig. 3, respectively. To quantify the growth of the newly formed bone around the two types of implants, the dynamic histomorphometric indices, that is, the MAR, are presented in Table 3 and Fig. 4. The data on the MAR obtained from computer image analysis are presented as the mean ± standard deviation (SD). As presented in Table 3 and Fig. 4, the MAR in the MAO group was greater than in the SF group, and there were statistically significant differences between the two groups at each observation period (Student’s unpaired *t*-test). The change in MAR in both groups was consistent: it increased from week 4 until week 8, when it reached its peak, and then it began to decrease until week 12, thus in the end having a slower speed than at week 4.

**Table 3:** The mineral apposition rate between the two groups at each observation period (μm·d^−1^)

Group (*n*=24)	Week 4	Week 8	Week 12
SF group	1.14±0.13	1.38±0.15	0.76±0.06
MAO group	1.35±0.10	1.45±0.20	0.93±0.07
*P*-value	*P* < 0.05	*P* < 0.05	*P* < 0.05

The rate in the MAO group was greater than in the SF group and had statistical significance in each observation period (Student’s unpaired *t*-test). *MAO* microarc oxidation, *SF* smooth surface

### Histological Examination

**Figure 5:**
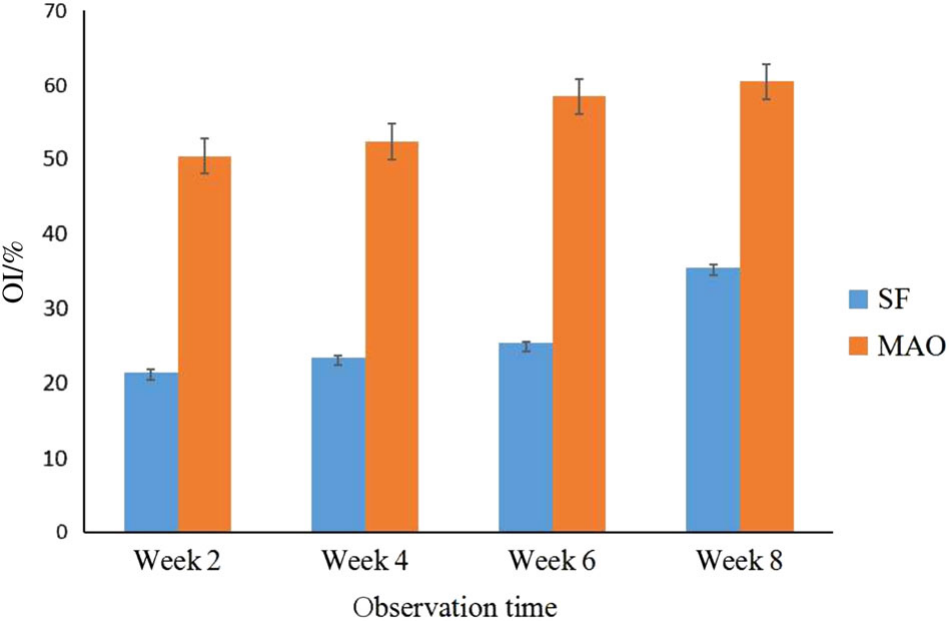
Osteointegration index (OI) values of the SF and MAO groups at different observation times. OI values of different groups at the same observation time show significant differences (*P*<0.05). MAO microarc oxidation, SF smooth surface

Samples were available for histological and histomorphometric analyses. Calcified bone matrix was recorded as red, osteocytes were seen as black spots, osteoids appeared blue–green, osteoblasts appeared dark blue, and fibrous tissue around implants presented as blue. Although both groups had new peri-implant bone formation in bone marrow regions at 2, 4, 8, and 12 weeks, the MAO-treated samples exhibited better and faster osseointegration at the implant–bone interface than the SF samples. The data on osteointegration index (OI) obtained from computer image analysis are presented as the mean ± SD. As indicated in Table 4, the OI values were greater in the MAO groups than in the SF groups. Student’s *t*-test revealed that the differences throughout the experiment (2, 4, 8, and 12 weeks postoperatively) were very statistically significant in both groups, indicating enhancement of bone formation around MAO-treated Ti implants in the local bone over time. Histograms of OI values at different observation times are shown in Fig. 5.

**Table 4:** Osteointegration index (OI) values of the SF and MAO groups at different observation times

	Week 2	Week 4	Week 8	Week 12
SF group	21.45 ± 0.20	23.36 ± 0.19	25.32 ± 0.38	35.45 ± 0.63
MAO group	50.45 ± 0.35	52.43 ± 0.28	58.45 ± 0.24	60.45 ± 0.42
*t*-value	−4.61	−3.28	−5.12	−5.63
*P*-value	*P* < 0.05	*P* < 0.05	*P* < 0.05	*P* < 0.05

OI values were greater in the MAO group than in the SF group; Student’s unpaired *t*-test showed that the difference was statistically significant with *P* < 0.05. *MAO* microarc oxidation, *SF* smooth surface

#### Week 2

**Figure 6:**
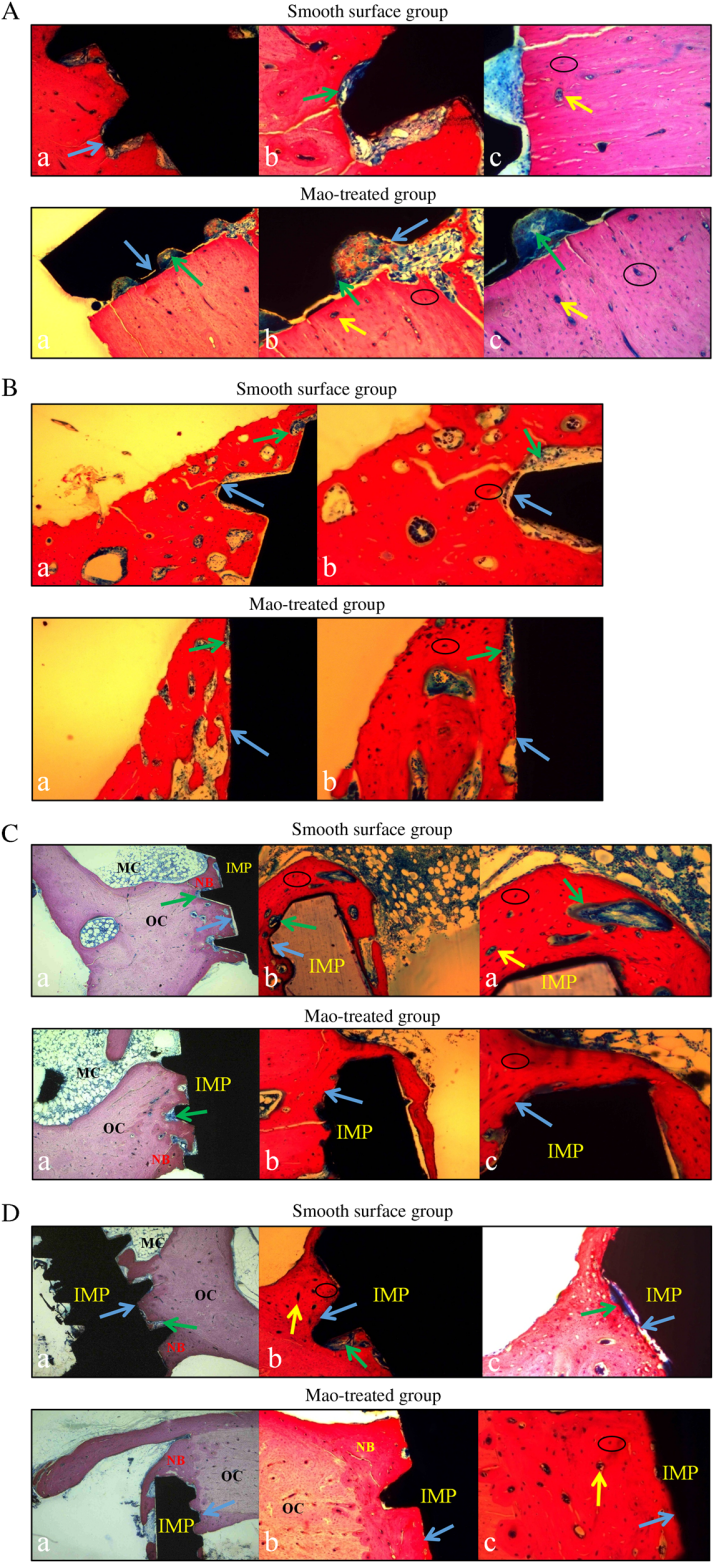
Histological examination of two groups at different times. Black circles indicate bone lacunas; yellow arrow represents Haversian system; green arrows indicate osteoblasts; blue arrows indicate bone-to-implant interface. NB means new born bone, OC represents original bone tissue, IMP means implant, and MC indicates bone marrow. A: Week 2. B: Week 4. C: Week 8. D: Week 12. **a** using magnification of 100 times, **b** using magnification of 200 times, **c** using magnification of 400 times

In the SF group, newly formed bone showed sparse contact with implants, and osteoclasts absorbed bone matrix, with many defects left Fig. 6a. A few osteoblasts appeared on the concave surfaces of the implant screws, and many osteocytes disappeared, leaving empty lacuna. A few Haversian systems existed and were far away from the implants. More blue osteoblasts predominated around the implant sites than red calcified bone matrix and black spot osteocytes.

In the MAO group, more blue osteoblasts were observed at the interface between the implant site and the original bone, and pink osteoids began to appear. No osteoclasts or absorbing areas, which were already filled with osteoblasts and new formed bone matrix, were seen. The viability of both the newly formed and original bone was demonstrated by the presence of osteocytes entrapped in the lacunae inside the bone trabeculum. More Haversian systems existed on the bone–implant interface, facilitating further osteointegration.

#### Week 4

In the SF group, the bone formation process occurred multicentrically Fig. 6b. Bone defects at the bone–implant interface obviously decreased, indicating that bone formation gradually replaced bone resorption. The number of osteoblasts on the concave surfaces of the implant screws was greater than in the SF group at week 2, but they were located mainly at the original bone site instead of at the implant site. Spot osteocytes could be observed in non-contact areas. There were obvious gaps between the bone and implant, suggesting that distant osteogenesis predominated.

In the MAO group, the osteogenic efficiency of the MAO group at week 4 was higher than in the control group. Calcified bone matrix attempted to fill most of the gaps between the implants and the original bones. Increasingly entrapped osteocytes were noted inside these lacunae, and dense osteoblasts were observed both on the bone side and on the implant side, revealing that distant osteogenesis occurred, coupled with contact osteogenesis.

#### Week 8

In the SF group, evident tissue differences in contrast with the SF group at week 4 were demonstrated by augmented calcified bone matrix, osteocytes embedded in lacunae and the density of the osteoblasts Fig. 6c. More calcified matrix was noted in the vicinity of the implants, while osteoblasts in the contact area were in the majority. Distant osteogenesis still predominated with apparent gaps between bones and implants.

In the MAO group, bone tissue contacted closely with implants without no obvious gaps. Compared with the groups at week 4, obvious differences were manifested in abundant calcified matrix in the vicinity of the implants, especially in the contact areas. Compared with the control group at week 8, calcified bone matrix and osteoids increased. Contact osteogenesis was confirmed by the presence of numerous entrapped osteocytes within this osteoid tissue and more intensive osteoblasts bordering the implant site, denoting that the effect of contact osteogenesis was superior to that in the control group.

#### Week 12

In the SF group, at low magnification, bone tissue neighbouring implants became mature with dark red stains, and massive newly formed bone and clear Haversian systems could be observed Fig. 6d. The amount of bone matrix and numbers of osteocytes and osteoblasts were larger than for the group at week 8. At high magnification, however, some blue osteoblasts still existed at the bone–implant interface, revealing a distant, osteogenesis-based bony process.

In the MAO group, bone near implants was almost calcified, and evident Haversian systems were noted. Dotted osteocytes and osteoids were visible in some regions, and no large osteoblast masses could be detected. The proportion of bone matrix to osteoblasts was significantly higher than in the groups at week 8 and in the SF group at week 12. Newly formed bone, appearing in the form of red-stained bone matrix without blue osteoblasts, contacted closely with implants, demonstrating that the effect of contact osteogenesis preceded that in the control group at the same time and preceded that in the MAO group at week 8.

### Comparative observations: fluorescence and light microscopic examinations

Comparative observations were conducted in the same area of one sample to determine the types of cells that exhibited more activity in the same observation period.

#### Week 4

**Figure 7:**
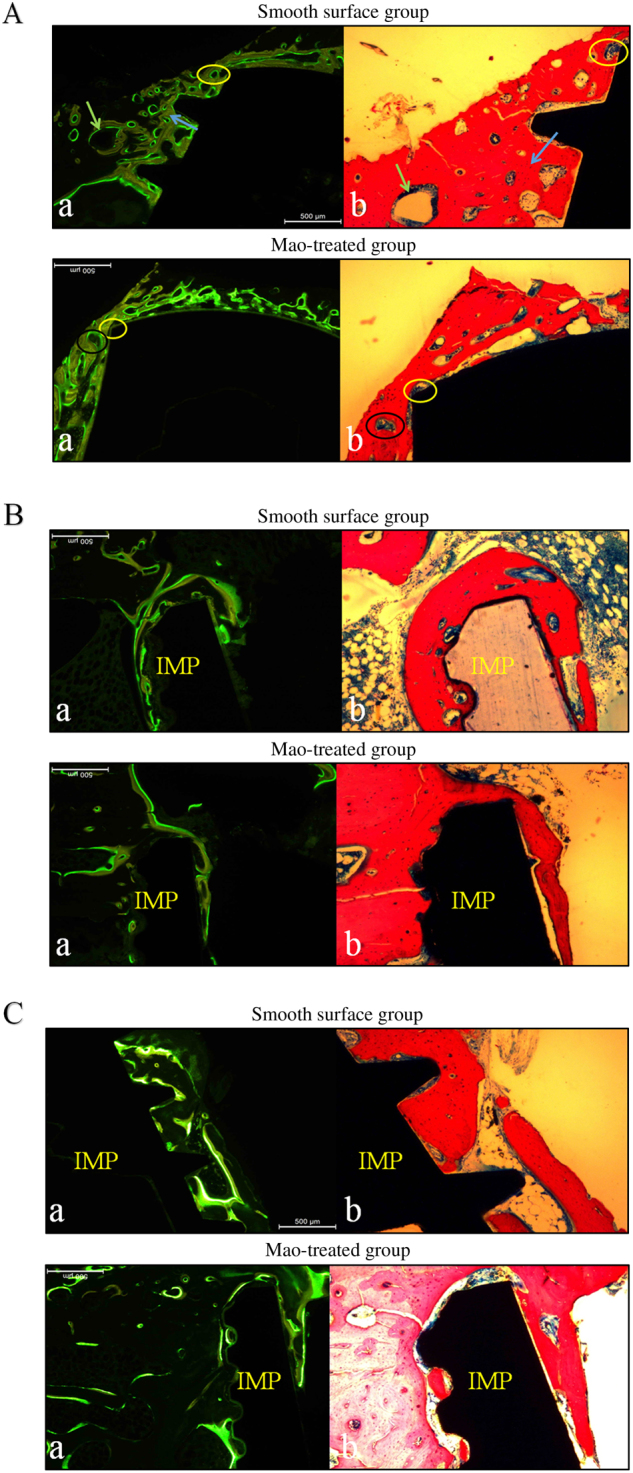
Comparative observations of two groups at different times. A: Week 4. B: Week 8. C: Week 12. **a** fluorescence microscopic examination, **b** light microscopic examination. IMP implant

In the SF group, three observation areas were selected as markers Fig. 7a. Yellow areas stained by tetracycline under fluorescence microscopy corresponded to red mature bone matrix under light microscopy (indicated by the blue arrow). Green areas stained by calcein under fluorescence microscopy coincided with deep blue osteoids under light microscopy (indicated by the green arrow). A cavity marked by a yellow circle under fluorescence microscopy was equated with blue osteoblasts under light microscopy. Only newly formed bone could be fluorescently labelled as a green area.

In the comparison of the two slices, the area labelled with fluorescent green denoted new growth districts of osteoblasts, while the fluorescent yellow area indicated gradually calcified bone matrix. No fluorescence was detected in the absorbing area of osteoclasts. It is important to note that fluorescent green strips were located in the original bone around the implant site, accounting for distant osteogenesis.

In the MAO group, fluorescent yellow strips constituted a proportion in contact with implants, demonstrating that osteogenesis in this area was already accomplished gradually at week 4. A non-fluorescent zone marked by a black circle under fluorescence microscopy was confirmed as osteoclasts by light microscopy at high magnification, and a bi-colour fluorescent zone corresponded to red bone matrix. A fluorescent green strip was detected in the bone-to-implant contact area, denoting that viable osteocytes bordered the implant site and grew towards the original bone, showing that osteogenesis presented bidirectionally. Compared with the control group, bone absorption was usual, while bone formation was relatively frequent.

#### Week 8

In the SF group, osteogenesis in part of the contact zone was almost finished at this time, the unlabelled area under fluorescence microscopy was filled with red mature bone matrix under light microscopy, and blue osteoblasts were mainly shown in evident fluorescent bands Fig. 7b.

In the MAO group, few fluorescent bands could be detected in the contact area, illustrating that osteointegration was substantially completed. Fluorescent areas were observed in the corresponding areas of blue osteoblasts. In contrast with groups at week 4, the interactive conditions of the two colour strips were invisible.

#### Week 12

In the SF group, fluorescent green strips persisted towards some contact areas, demonstrating that unidirectional and distant osteogenesis in this area predominated, which the fluorescence labelled mainly as blue osteoblasts Fig. 7c. The bone formation process was ongoing with osteointegration incomplete.

In the MAO group, osteointegration was fundamentally completed at this time point; fluorescent green strips developed towards the lateral aspect, and a less fluorescent area could be observed.

## Discussion

Advanced implants with improved osteointegration properties are always clinically in demand. Due to the non-degradability of Ti implants, their interactions with the cellular proceed only at their interfaces.^38^ It was demonstrated that MAO films possesses a powerful ability for vertical osteoconduction.^39^ Therefore, MAO has become a promising technique for surface modifications. In the present study, cellular responses, histological examinations, MARs and osteointegration indices were evaluated horizontally and vertically, and MAO-treated implants showed potent and rapid osteointegrative ability. This finding supports the great success of the surface modification technique in implant dentistry from a different point of view.

In horizontal comparisons between the MAO group and the SF group at the same observation time, the advantages of MAO modification were exhibited in the following three aspects: the mode of osteointegration, the rapid processes of bone remodelling and the high MAR. MAO-treated implants exhibited more active contact osteogenesis, as well as distant osteogenesis. There are two ways in which osteogenesis occurs: distant osteogenesis and contact osteogenesis.^40^ In contact osteogenesis, new bone forms on the implant surface, while in distant osteogenesis, the bone grows from the old bone surface towards the implant surface in an appositional manner. Contact osteogenesis can lead to bone bonding if the surface of the implant displays the appropriate surface topography.^41^ The phenomena of distant and contact osteogenesis can barely be observed in sections with ordinary staining; a fluorescent tracer technique was therefore adopted in our experiments to explore the processes of osteogenesis.

Recent studies have shown that osteoblasts produce intra- or extracellular amorphous Ca phosphate, indicating that early bone mineral formation is controlled by osteoblasts.^42^ Fully differentiated osteoblasts secrete collagen to form bone matrix and move backward at the same time, away from the advancing mineralization front; however, they are sometimes unable to escape and become enveloped.^43^ When this process occurs, the osteoblasts become osteocytes inside bone lacunae, and the formation of immature woven bone results, proceeding in an appositional manner from the surface of the implant to the cut edges of the bone. This process is called ‘contact osteogenesis’. Formation of new bone can also occur in the opposite direction, proceeding from the cut bone surface to the implant. This process is known as ‘distant osteogenesis’.^44^ In distant osteogenesis, the osteocytes within the cut bone edges die due to thermal necrosis during implantation, and the dead bone is absorbed by the osteoclasts.^45,46^ Differentiating osteoblasts migrate to the surface of the absorbed bone and form a noncollagenous cement line similar to that on the implant surface.^43^ Mineralization occurs from the cement line and into the collagen layer. Woven bone is produced by apposition, which extends from the cut bone surface to the implant surface. Thus, bone formation occurs in two opposite directions. Fluorochrome labelling of the bone suggests that contact osteogenesis occurs at a rate that is 30% faster than distant osteogenesis.^45,47^ Contact osteogenesis and distant osteogenesis result in immature woven bone formation around the implant. Secondary stability of the implant can arise from bone bonding.^44^

Ca- and P-containing oxide films are more beneficial for initial cell attachment and proliferation, and they can induce higher osteoconduction. The MAO technique generates a new nanocomposite-graded Ti oxide structure doped with Ca and P. The surface topography of implants modified by MAO is three-dimensionally complex, with pores and undercut coupled with the osteoconduction role of Ca and P, allowing for contact osteogenesis, and the cement line can interdigitate with the implant surface, leading to bone bonding. SF implants do not have the appropriate topography or favourable surface chemistry: the bone simply grows to the implant via distant osteogenesis, and bone bonding is not achieved.

The remodelling process is conducted by basic multicellular units through the interplay between osteoclastic and osteoblastic cell functions, leading to bone resorption or bone gain.^48^ At two weeks after implantation, absorption of host bone in the SF group was continued by osteoclasts. Weak osteogenic activity was demonstrated by few osteoblasts at the contact area and numerous empty lacunae. In the MAO group, viable entrapped osteocytes were noted inside these lacunae, while more osteoblasts predominated around the implant sites, and osteoids occurred with osteoclasts undetected. These findings indicate that bone resorption was accomplished by osteoclasts completely, and bone gain began to occur. In subsequent weeks, MAO-treated implants maintained remarkable advantages in osteoblastic activity and new bone formation nonetheless. Therefore, MAO-treated implants showed more favourable patterns of bone remodelling, compared with SF implants. The results of the OI values exhibited statistically significant differences between the two groups at the same time (*P* < 0.05).

In most studies, the MAR is calculated through the measurement of the space between yellow strips labelled by tetracycline and green strips marked by calcein. This method is difficult to apply in practice because of different breadths, so vast data acquisition is needed to eliminate errors. The role of confocal microscope in dentistry and medicine is now widely established. The ability to create thin, high-resolution optical sections makes it a valuable device used in materials science and the biological sciences.^49^ The application of CLSM could increase further if the microscope can operate at high speeds and is used in combination with other optical devices. It is considered to be a versatile optical technique. In our experiments, excitatory green laser at wavelengths of 538–544 nm was innovatively employed to activate the specimens, and green areas labelled by calcein were selected. The MAR could thus be assessed accurately by measuring the width of green bands using a CLSM. The MARs of the MAO group at different observation times in this study were greater than those in the SF group. The results might possibly be attributed to the porous multipore topography with Ca and P incorporated providing a better material environment for cell bonding and survival and could be favourable for Ca phosphate formation. The porous and rough surface containing Ca and P fabricated by MAO could provide a larger, more compatible and positively inductive contact area for the adhesion of osteoblasts; after adhesion, the cells produce extracellular matrix (ECM) and cytoskeletal proteins and spread on the substrate, resulting in a cell-covered surface. Although there are some hypotheses to explain the mineralization process around ECM formation,^50–52^ recent studies have shown that osteoblasts produce intra-cellular or extracellular amorphous Ca phosphate, indicating that early bone mineral formation is controlled by osteoblasts.^42^ In addition to facilitating the adhesion of osteoblasts, Ca-incorporated implants exhibit stronger removal torque values and intensively mineralized osteoids on the interfaces of implants,^33^ as well as the acceleration of Ca phosphate formation.

In vertical comparisons, fluorescence coupled with light microscopic examination rendered the process of osteointegration intuitionally observable. Comparative observation through the two techniques provided us with a new method to trace osteogenesis and cell types—another innovative point of our experiments. In each group, samples were obtained for histological observation at the 2nd, 4th, 8th, and 12th weeks after surgery, and the process of osteointegration was surveyed. The MAO groups exhibited more active contact osteogenesis, as well as distant osteogenesis, compared with the SF groups, which only s distant osteogenesis. Two modes of osteogenesis proceeded in an appositional fashion from the implant’s surface to the cut edges of the bone, thus accelerating bone growth and enhancing bone bonding.

## Materials and methods

### Fabrication of MAO Coating

Pure Ti (TA4) implants (4.0 mm × 8 mm) were provided by the Weigao Biological Materials Co., Limited (Weihai, People’s Republic of China), and were consecutively deoiled in sodium hydroxide solution for 5 min and cleaned with deionized water. Prior to MAO treatment, all of the specimens were etched in a Kroll's reagent solution (2% HF, and 10% HNO_3_, in 88% H_2_O) for 10 min to remove the native oxide layer. MAO of the fixtures was conducted by self-developed MAO equipment in an aqueous electrolyte composed of 0.13 mol·L^−1^ calcium acetate (Ca(CH_3_COO)_2_·H_2_O) and 0.06 mol·L^−1^ sodium dihydrogen phosphate (NaH_2_PO_4_·2H_2_O). During the MAO process, the applied voltage, pulse frequency, duty cycle and duration time were set at 300 V, 700 Hz, 5% and 10 min, respectively, at a constant temperature of 35 °C. After each treatment, the samples were cleaned with distilled water under sonication and then were dried in air. All of the samples were sterilized by autoclaving prior to biological experiments.

### Surface Characterization

Surface morphology was observed through a scanning electron microscope (SEM, Zeiss EVO18, Zeiss Semiconductor Co., Ltd., Germany), and the chemical compositions of different surfaces were analyzed by INCA Energy dispersive X-ray analysis (EDS, Oxford Instruments Co., Ltd.).

Electric contourgraphs (FORM ALfYSUDF120, Precision Measuring Instrument Co., Ltd., Guangzhou, China) were applied for surface roughness analysis. Ra is presented as the mean deviation of the profile offset by the sampling length (Profile Arithmetical Deviation):

1 $${\mathrm{Ra}} = \frac{1}{{n}}\mathop {\sum}\limits_{{i} = 1}^{n} {\left| {{\mathrm{yi}}} \right|}$$

Both chemical elements analyzed by EDS and roughness measurements provided by electric contourgraphs were measured at three different points on every implant, and the means of the three measurements were calculated.

### Implantation

A total of 32 SF implants and 32 MAO-treated implants were prepared. The two different types of implants were separated into two groups, and each group was separated into four subgroups according to different durations of implantation. The experiment was conducted on a total of 16 New Zealand White female rabbits at the age of 5–6 months and weighing 2.5–3 kg. The animals were housed in separate cages in temperature-controlled rooms, were fed standard food and were given free access to tap water. The animals were cared for according to the guidelines of the local Ethics Committee of the Animal Research at Qingdao University, which approved the project before the beginning of the experiments. The rabbits were separated into four groups randomly according to implantation time, and the tibias of each rabbit had four implant sites that received different surface-treated implants randomly.

The rabbits were anaesthetised intramuscularly with pentobarbital sodium 30 mg·kg^−1^ body weight once general anaesthesia was established, the medial aspects around the proximal tibia were shaved, and the skin was carefully swabbed with a mixture of 2% iodine. A 1.5–2.5 cm incision was made along the medial aspect of the proximal tibia, and the wound advanced down to and through the periosteum. Subperiosteal dissection was then advanced up to the inferior attachment of the knee joint capsule and laterally to the full extent of the flat medial bone surface. Under continuous irrigation with 0.9% sterile saline at 4 °C, the implant installation procedure in the tibia was performed with rotation speed of 800 r·min^−1^ and depth maintained at 8 mm, and the implantation torque was ~30 N. The prophylactic administration of four million IU penicillin commenced after the surgery and continued for 3 days to reduce the potential for wound infection, and X-ray films were obtained.

### Fluorescence Labelling of Bone Specimen

Four groups of rabbits were killed in turn after 2, 4, 8, or 12 weeks through air embolism. Fluorescence labelling was conducted using tetracycline hydrochloride and calcein in the 4, 8, and 12 week groups (and not the 2-week group) before killing the rabbits. Calcein at a concentration of 20 mg·kg^−1^ body weight was administered to the rabbits by subcutaneous injection in physiological saline solution 3 and 4 days before the collection of specimens. At 13 and 14 days before the killing of animals, tetracycline in physiological saline was also administered by subcutaneous injection. Dissection of the operation area was performed after the rabbits were killed, and no fistula or abscesses were found.

### Manufacture of Bone Mill Chips

At 4, 6, and 12 weeks after implantation, the rabbits were killed and subjected to histological analysis. The tibia was dissected at 2 mm mesial and distal to the bone around the implant reserved, and the bone blots were fixed in formaldehyde solution for 7 days and then dehydrated in increasing grades of ethanol. The specimens were then embedded in polymethyl methacrylate resin and cut longitudinally along the centre of the long axis, and each block was sectioned with a high-precision diamond disk at ~200 μm in thickness and was ground to ~40 μm in final thickness using an Exakt 400 CS grinding device.

### Histological Examination

**Figure 8:**
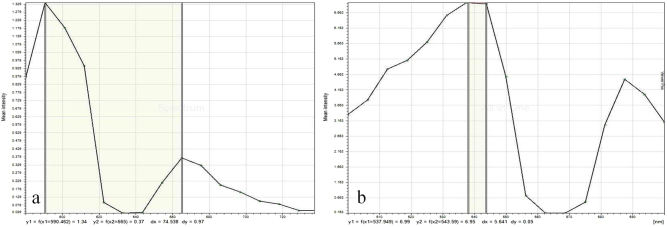
Excitation values of red and green lasers. **a** Activated by rad laser, **b** activated by green laser

Light and fluorescence microscopic examination (Olympus, Japan) were performed for tracing observation of tetracycline-calcein labelling in specimens. The specimens regularly treated with tetracycline showed fluorescence of yellow strips after activation by green light, while calcein was shown in green strips. This effect was applied to show newly formed bone (green fluorescence) more clearly in contrast to yellow fluorescence. The samples were then examined under a CLSM (Olympus, Japan). Excitation values of red and green laser were tested, and excitation peaks are shown in Fig. 8. Red laser at 598–664 nm wavelength was first applied to activate the specimen; the areas labelled by tetracycline and calcein, however, could not be distinguished obviously, and the precise calcification rate could not be calculated accordingly. Excitation green laser at a wavelength of 538–544 nm was then employed to activate the specimen, and a green area labelled by calcein was selected. The mineral apposition rate (MAR) was assessed accurately by measuring the width of green bands using Image-pro Plus software, version 6.0, applied to CLSM images. After examination by CLSM, the samples were stained by acid fuchsine-methylene blue to evaluate the osteointegration at the bone–implant interface. Calcified bone matrix was recorded as red, osteocytes were seen as black spots, osteoids appeared blue–green, osteoblasts appeared dark blue, and fibrous tissue around implants presented as blue under a light microscope. Images were transformed into data. The OI was calculated by the following formula:

2 $${\mathrm{OI}} = \frac{{{\mathrm{the}}\,{\mathrm{length}}\,{\mathrm{of}}\,{\mathrm{osteointegration}}}}{{{\mathrm{the}}\,{\mathrm{length}}\,{\mathrm{of}}\,{\mathrm{bone - implant}}\,{\mathrm{interface}}}} \times 100{\mathrm{\% }}$$

### Statistical Analysis

Statistical analysis was performed using SPSS software, version 16.0. The data obtained from computer image analysis are presented as the mean and standard deviation and were tabulated and statistically analyzed. Student’s *t*-test was used for statistical analysis of the differences between groups. A *P-*value <0.05 was considered statistically significant.

## Conclusions

In our study, the process of bone–implant integration was evaluated using light, fluorescence and confocal laser scanning microscopy, and the main conclusions are as follow.

1. MAO-treated implants offer topographic surface features rich in Ca and P ions and can enhance osteoconduction and the resulting bone bonding, possessing good osteointegration ability.
2. The excellent osteointegration ability of MAO-treated implants is reflected in more active contact osteogenesis and a quicker mineral apposition rate.

This study offers a meaningful theoretical basis for clinical application and early loading of MAO-treated Ti implants, and it could also provide a reference for other types of molecule delivery from implant surfaces, shedding new light on present studies. Further studies involving more in vitro tests are currently in progress, and they might provide more evidence showing that a MAO-treated porous surface layer with desirable process parameters contributes a good microenvironment for cell survival and growth. The bone–implant integration mechanisms and the interactions between osteogenic cells and implant surface charges should be of particular interest.
